# Exploring the association and causal effect between white blood cells and psoriasis using large-scale population data

**DOI:** 10.3389/fimmu.2023.1043380

**Published:** 2023-02-14

**Authors:** Guowei Zhou, Xiangmei Ren, Zhenwei Tang, Wang Li, Wenqiong Chen, Yi He, Benliang Wei, Hailun Zhang, Fangyu Ma, Xiang Chen, Guanxiong Zhang, Minxue Shen, Hong Liu

**Affiliations:** ^1^ Department of Dermatology, Xiangya Hospital, Central South University, Changsha, Hunan, China; ^2^ Hunan Key Laboratory of Skin Cancer and Psoriasis, Xiangya Hospital, Changsha, Hunan, China; ^3^ Hunan Engineering Research Center of Skin Health and Disease, Xiangya Hospital, Changsha, Hunan, China; ^4^ Department of Research and Development, Beijing GAP Biotechnology Co., Ltd, Beijing, China; ^5^ Department of Health Management Center, Xiangya Hospital, Central South University, Changsha, Hunan, China; ^6^ National Clinical Research Center for Geriatric Disorders, Xiangya Hospital, Central South University, Changsha, Hunan, China; ^7^ Department of Social Medicine and Health Management, Xiangya School of Public Health, Central South University, Changsha, Hunan, China

**Keywords:** psoriasis, white blood cells, Mendelian randomization, neutrophil-lymphocyte ratio, platelet-lymphocyte ratio, lymphocyte-monocyte ratio

## Abstract

**Introduction:**

Psoriasis is a chronic inflammatory disease of the skin. A few studies have shown that psoriasis is an immune-mediated disease in which multiple immune cells play crucial roles. However, the association between circulating immune cells and psoriasis remains elusive.

**Methods:**

To explore the role of circulating immune cells in psoriasis, 361,322 individuals from the UK Biobank (UKB) and 3,971 patients with psoriasis from China were included to investigate the association between white blood cells and psoriasis *via* an observational study. Genome-wide association studies (GWAS) and Mendelian randomization (MR) were used to evaluate the causal relationship between circulating leukocytes and psoriasis.

**Results:**

The risk of psoriasis increased with high levels of monocytes, neutrophils, and eosinophils (relative risks and 95% confidence intervals, respectively: 1.430 (1.291–1.584) for monocytes, 1.527 (1.379–1.692) for neutrophils, and 1.417 (1.294–1.551) for eosinophils). Upon further MR analysis, eosinophils showed a definite causal relationship with psoriasis (odds ratio of inverse-variance weighted: 1.386, 95% confidence intervals: 1.092–1.759) and a positive correlation with the psoriasis area and severity index (PASI) score (*P* = 6.6 × 10^-5^). The roles of the neutrophil-lymphocyte ratio (NLR), platelet-lymphocyte ratio (PLR), and lymphocyte-monocyte ratio (LMR) in psoriasis were also assessed. More than 20,000 genetic variations associated with NLR, PLR, and LMR were discovered in a GWAS analysis using the UKB data. Following adjustment for covariates in the observational study, NLR and PLR were shown to be risk factors for psoriasis, whereas LMR was a protective factor. MR results indicated that there was no causal relationship between these three indicators and psoriasis; however, NLR, PLR, and LMR correlated with the PASI score (NLR: rho = 0.244, *P* = 2.1 × 10^-21^; PLR: rho = 0.113, *P* = 1.4 × 10^-5^; LMR: rho = -0.242, *P* = 3.5×10^-21^).

**Discussion:**

Our findings revealed an important association between circulating leukocytes and psoriasis, which is instructive for the clinical practice of psoriasis treatment.

## 1 Introduction

As a common chronic inflammatory skin disease, psoriasis affects more than 60 million adults and children, considerably impairs the quality of life of patients, and places a heavy burden on individuals and society (1); however, the pathogenesis of psoriasis has not yet been fully elucidated (2). Immune cells play a crucial role in the pathogenesis of psoriasis (1, 3, 4), and previous studies have indicated the importance of systemic immunity in psoriasis, such as excessive interleukin 17 (IL-17) and interleukin 36, in circulating immune cells (5, 6). Meanwhile, patients with psoriasis have shown a unique profile of circulating leukocytes (7, 8), and increased neutrophil counts have been discovered in multiple studies (9, 10). However, more studies have focused on immunocytes from the skin lesions of psoriasis (11–13), and the role of circulating white blood cells in psoriasis remains unclear. Lymphocytes, especially T cells, have been recognized as disease-causing cells in psoriasis (14); however, some studies have shown decreased circulating lymphocytes in patients with psoriasis (15). The relationship between psoriasis and other circulating cells, such as eosinophils and basophils, has been rarely reported. Therefore, it is necessary to explore this association further.

Although some studies have shown the important role of circulating immune cells in psoriasis, these results were based on observational studies that were limited by sample size and bias. More appropriate methods are required to improve these studies. Mendelian randomization (MR) studies use instrumental variables associated with exposure to assess possible causal relationships with outcomes. This method can reduce the potential confounding bias (16, 17). The causal relationships between various phenotypes and diseases have been revealed using MR analysis (18, 19). Thus, it is feasible to use MR to explore the causal relationship between circulating immune cells and psoriasis.

To further understand the role of circulating immune cells in psoriasis, we explored the association between five main types of white blood cells and psoriasis using data from the UK Biobank (UKB) and a Chinese population and evaluated the potential causal relationship between psoriasis and white blood cells. We further explored the roles of the neutrophil-lymphocyte ratio (NLR), platelet-lymphocyte ratio (PLR), and lymphocyte-monocyte ratio (LMR) in psoriasis. Our results provide a comprehensive overview of the association between circulating white blood cells and psoriasis, suggesting that eosinophil count is a potential factor related to the incidence and severity of psoriasis.

## 2 Methods

### 2.1 Study population

**Figure 1:**
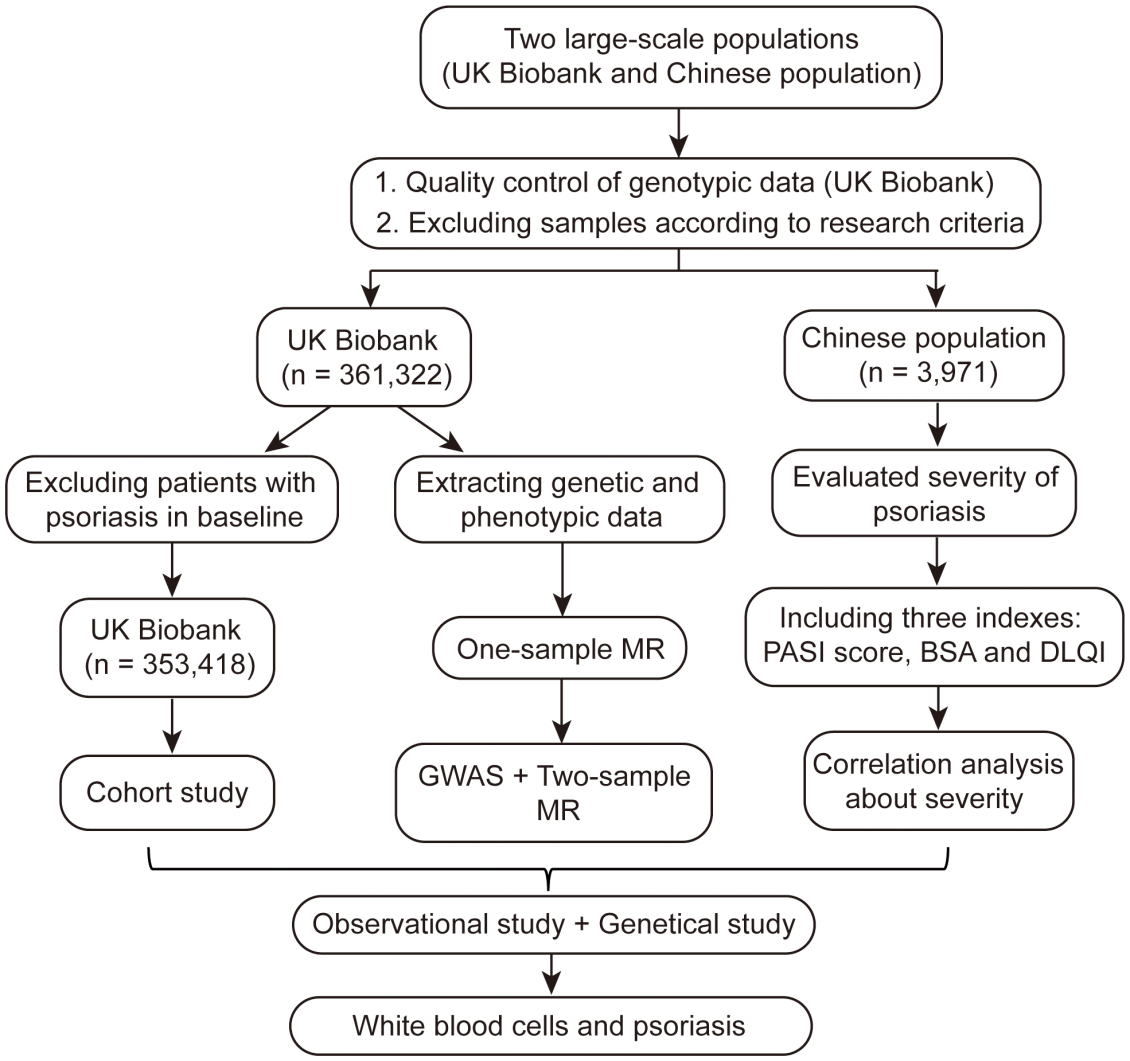
Flowchart of the study about white blood cells and psoriasis.

In total, 361,322 individuals from the UKB and 3,971 individuals from a Chinese population were included in our study (**Figure 1**). The UKB is a prospective cohort study with a large amount of genetic and phenotypic data collected from approximately 500,000 individuals across the United Kingdom from 2006 to 2010 (20–22). Patients with psoriasis were included from primary care, hospital admission, self-reports, and other sources in the UKB (**Table S1**). Participants were excluded from the UKB dataset based on the following criteria: it did not pass quality control of genotypic data (missing information on individual data > 0.02, sex discrepancy, and deviates of more than ±3 standard deviations (SD) from the heterozygosity rate mean of the samples); kinship >first-degree relationship; genetic ethnic grouping showed non-Caucasian (defined by data-field: 22,006 from UKB based on a principal components analysis of the genotypes) (23); suffering from diseases of the blood and hematopoietic system including leukemia, lymphoma, multiple myeloma, aplastic anemias, and agranulocytosis, among others (**Table S1**); and a white blood cell count >200 × 10^9^ cells/L (24). The Chinese population included patients with psoriasis admitted to Xiangya Hospital, Central South University between 2019 and 2020. Patients with diseases of the blood and hematopoietic system and abnormal white blood cell count (>200 × 10^9^ cells/L) were excluded. The diagnosis of psoriasis in the Chinese population was confirmed by two or more dermatologists. The UKB received ethical approval from the Northwest Multi-Center Research Ethics Committee (11/NW/03820). All procedures involving study participants in the Chinese population were approved by the institutional research ethics board of Xiangya Hospital (2018121106). Written informed consent was obtained from all participants prior to the investigation.

### 2.2 Exposure and outcome variables

We selected white blood cells, lymphocytes, monocyte, neutrophil, eosinophil, and basophil counts as indicators of circulating leukocytes (19). All measured data of white blood cells were sourced from the baseline in the UKB. To obtain the white blood cell count, the Beckman Coulter LH750 instruments were used to analyze blood samples collected in EDTA (Ethylenediaminetetraacetic acid) vacutainers from participants in the UKB and Chinese populations. Regarding data distribution, eosinophils, and basophils showed a skewed distribution in the UKB (**Figure S1**). Moreover, the five main white blood cell types correlated with each other (**Figure S2**, all *P <*2.2 × 10^-16^). We simultaneously calculated the NLR, PLR, and LMR *via* neutrophil count, lymphocyte count, monocyte count, and platelet count excluding individuals whose denominator was zero.

The phenotype of psoriasis was identified using the codes of the International Classification of Disease (ICD-9 and ICD-10) for participants in the UKB and Chinese population. We evaluated the severity of psoriasis using three indices: psoriasis area and severity index (PASI) score, dermatology life quality index (DLQI), and body surface area (BSA) (25–27). The PASI score was calculated based on the intensity of three clinical signs (redness, thickness, and scaling) in four sections of the body (head, arms, trunk, and legs) (more details are available at https://dermnetnz.org/topics/pasi-score). The BSA was calculated based on the proportion of skin affected by psoriasis, denoted as a percentage of the total body area. The DLQI was determined *via* questionnaires to assess the extent to which the skin problem has affected the patients’ lives over the last week (questionnaires are available at https://www.nhsfife.org/media/32589/dermatology-life-quality-index-dlqi.pdf). Dermatologists evaluated the PASI score and BSA according to the area and severity of lesions, and the DLQI was obtained from a questionnaire.

### 2.3 Observational study

Patients with psoriasis at baseline were excluded from the UKB, and 353,418 participants were included in the cohort study. The mean follow-up time was 12.88 years. Z-score standardization was adopted to process the data to eliminate the interference of different dimensions among the types of white blood cells. White blood cell count and the count of the five main types of white blood cells were divided into four categories in the UKB based on quartiles. Relative risks (RRs) and 95% confidence intervals (95% CIs) were calculated for each category, using the first category as a reference. We also analyzed the relationship between different types of white blood cells and psoriasis in continuously defined white blood cell counts using Cox regression models with age as the time scale and logistic regression models. Sex, age, smoking status (never, previous, and current), alcohol drinking status (never, previous, and current), and body mass index (BMI) were adjusted as covariates (28). A sensitivity analysis excluding individuals with a reported incidence of psoriasis in the first two years after baseline was performed to confirm the findings. Owing to the correlations among the main types of white blood cells (**Figure S2**), the lasso regression model, including five white blood cell types and covariates, was used to select important variables and avoid potential multicollinearity (29). For the NLR, PLR, and LMR, z-score standardization was used to remove the impact of dimension, and the association with psoriasis was evaluated using the Cox regression model. In the Chinese population, Spearman’s correlation was used to determine the correlation between indices associated with white blood cells and severity in a total of 3,971 patients with psoriasis. The PASI score, DLQI, and BSA were all included in the correlation study.

### 2.4 One-sample MR analysis

To test the causal relationship between white blood cells and psoriasis, a one-sample MR analysis was performed in the UKB. First, 482, 457, 512, 372, 464, and 144 single nucleotide polymorphisms (SNPs) associated with white blood cell, lymphocyte, monocyte, neutrophil, eosinophil, and basophil counts, respectively, were selected from a meta-analysis (GWAS) study (**Table S2**) (30). These SNPs were used as instrumental variables after deleting ambiguous SNPs and controlling for linkage disequilibrium (LD, reference panel: European from the 1000 genomes reference panel, r^2^ <0.01, window: 10,000 kb): 178 SNPs for white blood cell count, 140 SNPs for lymphocyte count, 167 SNPs for monocyte count, 128 SNPs for the neutrophil count, 166 SNPs for eosinophil count, and 51 SNPs for basophil count (23). The genetic risk score (GRS) of each sample in the UKB was calculated using the selected instrumental variables (31). Allele scores were weighted based on the effect size in the meta-GWAS analysis (30). Thereafter, we evaluated the degree of variation explanation for GRSs and performed z-score standardization for GRSs. White blood cell, lymphocyte, monocyte, neutrophil, eosinophil, and basophil counts were explained by 2.66%, 1.76%, 3.00%, 1.94%, 3.34%, and 0.39%, respectively. The GRSs of white blood cells were divided into four categories according to quartiles. The calculated GRSs were separately associated with circulating immune cell counts (**Figure S3**). Finally, one-sample MR analysis was performed by two-stage least-squares regression (2SLS) to explore the causal relationship between white blood cells and psoriasis after adjusting for age, sex, BMI, and the top 10 genetic principal components (32). To remove the effect of potential pleiotropy, the association between possible confounding factors (sex, age, BMI, smoking status, and alcohol drinking status) and GRSs was further examined. For categorical variables (sex, smoking status, and alcohol drinking status), logistic regression models were constructed, and categorical variables were viewed as dependent variables to test the association with the count and GRSs of white blood cells. For continuous variables (age and BMI), the count and GRSs of white blood cells were divided into four categories according to quartiles, and the difference in continuous variables was compared among all categories using the Kruskal–Wallis test (32). Thereafter, we performed MR analysis again after excluding SNPs related to confounding factors using PhenoScanner v2 (**Table S3**) (33).

### 2.5 Two-sample MR analysis

Two-sample MR analysis was conducted to further verify the results from one-sample MR. Two GWAS summary statistic datasets from MRC-IEU (34, 35) (https://gwas.mrcieu.ac.uk/) and the FinnGen biobank (https://www.finngen.fi/en) were used to select SNPs as instrumental variables. The dataset related to white blood cells was obtained from the study by Astle et al. (18), and the other dataset related to psoriasis was obtained from the FinnGen biobank (36) (release: R2, finn-a-L12_PSORIASIS, and the phenotype of psoriasis were identified by ICD-8, ICD-9, and ICD-10). Final analyses included significant genome-wide (*P <*8.31 × 10^-9^) and uncorrelated SNPs (r^2^ <0.01) available in GWAS summary data of exposure variables. Seven methods of MR analysis were used to perform two-sample MR, including inverse-variance weighted (IVW) (37), MR-Egger (38), weighted median (39), simple mode (40), weighted mode (40), MR-PRESSO (41), and multivariable MR (42). The heterogeneity of all instrumental variables was determined using Cochran’s Q test. Steiger filtering tests were used to filter SNPs related to psoriasis, and Egger intercept tests were performed to check the pleiotropy of instrumental variables. Leave-one-out sensitivity analyses were performed after two-sample MR (43). To further explore causal relationships between relative indicators (NLR, PLR, and LMR) and psoriasis, we performed GWAS analysis for these three indicators in UKB. By setting the threshold of the *P* value, we discovered 5973 SNPs associated with NLR, 15473 associated with PLR, and 4606 associated with LMR (*P* adjusted by false discovery rate <8.31 × 10^-9^, **Figure S4**). Thereafter, SNPs associated with these three indicators (reference panel: European from the 1000 genomes reference panel, r^2^ <0.01, window: 10,000 kb) in our GWAS results and summary data associated with psoriasis from the FinnGen biobank were used to perform a two-sample MR analysis.

### 2.6 GWAS analysis

Genotype data in the UKB were obtained from imputation using HRC and UK10K as reference samples (21, 44). NLR, PLR, and LMR were used as phenotypic data to perform the GWAS. Quality control was completed before association analysis (see Section 2.1; missing information on SNPs < 0.02; minor allele frequency <0.05 and Hardy–Weinberg equilibrium <1 × 10^-6^ were used to filter SNPs to avoid the rarity and instability of SNPs) (45). The GWAS analysis was adjusted for age, sex, BMI, and the top 10 principal genetic components (24, 30). To distinguish confounding factors from polygenicity in our GWAS study, we used LD score regression to calculate the LD score regression intercepts (46). The intercepts of NLR, PLR, and LMR were 1.09, 1.12, and 1.08, respectively, which showed low confounding biases such as cryptic relatedness and population stratification. The source of the LD score was from Europe in the 1000 Genomes Project, and LD score regression was performed using ldsc (https://github.com/bulik/ldsc) (46).

### 2.7 Statistical analysis

We used R language (version 4.0.5) to analyze the data. GWAS was performed using PLINK v1.9. The “AER” package in R was used in one-sample MR. GRSs were calculated using PRSice-2 (47). The “TwoSampleMR” and “MRPRESSO” packages were used to perform two-sample MR and sensitivity analyses (34).

## 3 Results

A total of 361,322 individuals from the UKB and 3,971 individuals from the Chinese population were included in our study. The characteristics of all included individuals are shown in **Table 1**.

**Table 1:** Characteristics of participants in UKB and Chinese population.

Characteristic	UK Biobank (n = 361,322)	Chinese population (n = 3,971)
**Sex,%**		
male	46.50	62.83
female	53.50	37.17
**Age in years, Mean**	56.84	40.60
**Smoking status, %**		
Never	54.49	57.47
Previous	35.04	7.83
Current	10.12	30.60
Missing^1^	0.35	4.10
**Alcohol drinker status, %**		
Never	3.00	65.02
Previous	3.31	11.58
Current	93.61	19.42
Missing^1^	0.08	3.98
**Body Mass Index, Kg/m2, Mean**	27.39	26.94
**White blood cell count, 10^9^ cells/Litre, Mean**	6.87	7.23
**Lymphocyte count, 10^9^ cells/Litre, Mean**	1.94	2.00
**Monocyte count, 10^9^ cells/Litre, Mean**	0.48	0.54
**Neutrophil count, 10^9^ cells/Litre, Mean**	4.24	4.53
**Eosinophil count, 10^9^ cells/Litre, Mean**	0.17	0.19
**Basophil count, 10^9^ cells/Litre, Mean**	0.03	0.08
**Psoriasis^2^, %**	0.84	–

This study included 361,322 participants from UKB and 3,971 participants from Chinese population. ^1^Missing represented NA value; ^2^Psoriasis represented the incidence of psoriasis in UKB after excluding patients with psoriasis in baseline.

### 3.1 Association of white blood cells with psoriasis

**Figure 2:**
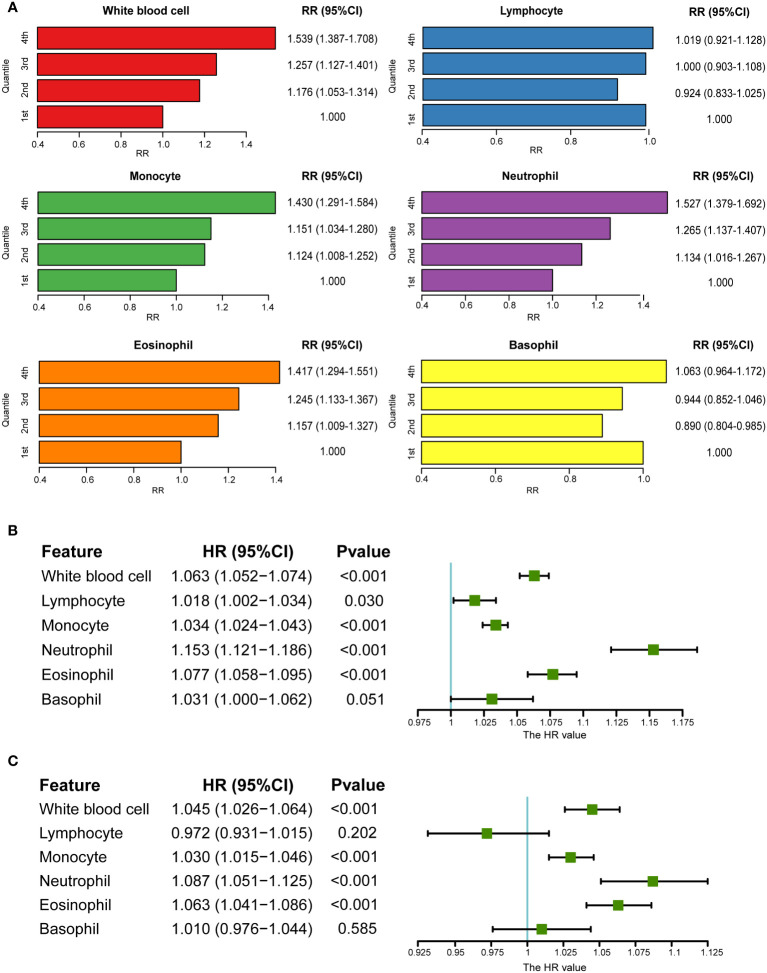
Association between circulating white blood cells and psoriasis in UKB. **(A)** RRs of psoriasis from categories of white blood cells (the first category was viewed as a reference). **(B)** Association between white blood cells and psoriasis in univariate Cox regression. **(C)** Association between white blood cells and psoriasis after adjusting for covariates in multivariate Cox regression (sex, age, BMI, smoking status, and alcohol drinking status).

Using the UKB data, a cohort study was performed after excluding patients with baseline psoriasis. The RRs of psoriasis incidence increased with the increase in monocytes (RR_1st vs_. _4th_: 1.430, 95% CI: 1.291–1.584), neutrophils (RR_1st vs_. _4th_: 1.527, 95% CI: 1.379–1.692), and eosinophils (RR_1st vs_. _4th_: 1.417, 95% CI: 1.294–1.551) (**Figure 2A**). This was further verified by the analysis of continuously defined white blood cell counts in Cox regression models. Hazard ratios (HRs) were calculated after introducing the time of psoriasis onset. Monocytes, neutrophils, and eosinophils were found to be risk factors for psoriasis (**Figures 2B, C**). HRs were calculated again after excluding individuals with a reported incidence of psoriasis in the first two years after baseline. Similar results were observed after adjusting for covariates (HR of monocytes: 1.033 (1.022, 1.044), HR of neutrophils: 1.147 (1.110, 1.184); HR of eosinophils: 1.076 (1.054, 1.097)) (**Table S4**). Furthermore, logistic regression models without consideration for time of incidence also showed consistent results (**Table S5**). Owing to the correlation among the five major white blood cell types (**Figure S2**), the lasso regression model was used for the five major white blood cell types and confounding factors. Lymphocytes, monocytes, neutrophils, and eosinophils were retained, and it was found that BMI and smoking status played an important role in psoriasis, which is consistent with the results of previous studies (28, 48) (**Figure S5**).

### 3.2 Causal effect of white blood cells on psoriasis

The above results show that there is an association between white blood cells and psoriasis. However, whether a causal relationship between them exists needed to be further explored. One-sample MR analysis was performed to explore the potential causal relationship by 2SLS in the UKB. The GRSs of white blood cells were selected as instrumental variables. The result of one-sample MR showed that eosinophils (OR: 1.032, *P* = 7.37 × 10^-3^) and basophils (OR: 1.545, *P* = 2.87 × 10^-6^) had a positive causal relationship with psoriasis after adjusting for covariates (**Table 2**).

**Table 2:** One-sample MR results of white blood cells and psoriasis in UKB. .

	Univariate 2SLS			2SLS with covariates		
	OR in psoriasis	SE	P	OR in psoriasis	SE	P
**White blood cell count**	0.998	0.001	9.31×10^-2^	0.998	0.001	9.03×10^-2^
**Lymphocyte count**	1.004	0.003	2.29×10^-1^	1.003	0.003	2.46×10^-1^
**Monocyte count**	0.993	0.008	3.74×10^-1^	0.993	0.008	3.37×10^-1^
**Neutrophil count**	0.998	0.001	1.59×10^-1^	0.998	0.001	1.53×10^-1^
**Eosinophil count**	1.034	0.012	5.01×10^-3^	1.032	0.012	7.37×10^-3^
**Basophil count**	1.537	0.093	3.69×10^-6^	1.545	0.093	2.87×10^-6^

The model of 2SLS was adjusted for age, sex, BMI and top 10 genetic principal components. The result of basophil count after deleting SNPs related BMI also show the causal relationship between basophil count and psoriasis. The OR and 95%CI for basophil count was 1.520 (1.266-1.826).

To determine potential pleiotropy, we tested whether confounding factors were associated with the GRSs of white blood cells. The results indicated that sex, age, smoking status, and alcohol drinking status were associated with the count of the five main types of white blood cells, and not with the calculated GRSs. Only BMI showed a correlation with the GRSs of total white blood cells and basophils (*P <*0.05), which might be a confounding factor causing pleiotropy in the MR analysis (**Figure S6**, **S7**, and **S8**). Considering the significant causal relationship between basophils and psoriasis (*P* = 2.87 × 10^-6^), SNPs associated with BMI were excluded from instrumental variables associated with basophils using PhenoScanner v2 (33), and one-sample MR analysis was performed again. The results showed that the causal relationship was still significant (OR: 1.520, 95% CI: 1.266–1.826).

### 3.3 Validation of causal relationship using two-sample MR

**Figure 3:**
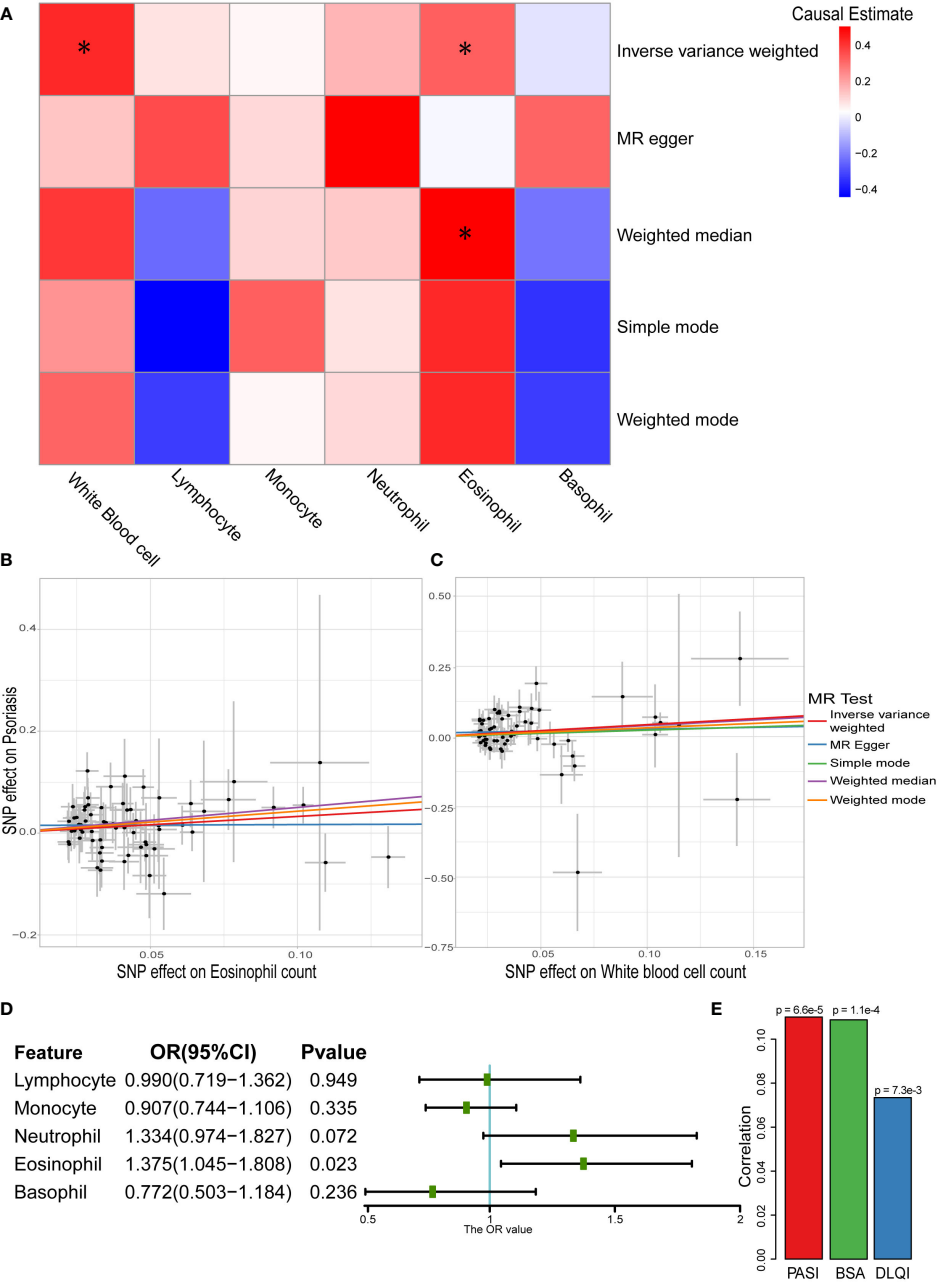
Causal relationship between white blood cells and psoriasis in two-sample MR analysis. **(A)** Heatmap of two-sample MR results about white blood cells and psoriasis (* represented *P* < 0.05). **(B)** Scatter plot of MR analysis related to eosinophil count and psoriasis. **(C)** Scatter plot of MR analysis related to white blood cell sum count and psoriasis. **(D)** Result of multivariable MR on different white blood cells and psoriasis. **(E)** Correlation between eosinophil count and severity of psoriasis (Spearman’s correlation).

According to the one-sample MR results, eosinophils and basophils showed a causal relationship with psoriasis. However, because of the existence of “winners’ curse,” the conclusion required more data sources to be verified (49). To further test this result, two-sample MR analysis was performed, and the results revealed that the total white blood cell count and eosinophil count had positive causal relationships with psoriasis (**Figures 3A-C** and **Table S6**). In particular, both IVW and weighted median showed a positive causal relationship between eosinophil count and psoriasis (OR_IVW_: 1.386, 95% CI: 1.092–1.759; OR_WM_: 1.654, 95% CI: 1.129–2.422), which was consistent with the results of the one-sample MR. To eliminate possible pleiotropy, MR-PRESSO was used to exclude outlier SNPs. We used 1,000 elements to form the null distribution for simulation to compute empirical *P*-values; only one outlier was discovered in monocytes. In addition, results of Egger intercept test also showed low pleiotropy of SNPs. Meanwhile, the heterogeneity test showed potential heterogeneity in lymphocytes and monocytes (*P* < 0.05) (**Table S6**). The random effects model further explored the causal effect of lymphocytes and monocytes, and the results indicated that there was still no significant causal relationship between them and psoriasis (lymphocytes: *P* = 0.6551; monocytes: *P* = 0.6788). The Steiger filtering test showed that all instrumental variables exerted their primary effect on circulating immune cells rather than psoriasis. To make the results more reliable, leave-one-out sensitivity analyses were performed, which did not identify any SNPs with a marked impact on the results (**Table S7**) (43).

Owing to the correlation among the five main types of white blood cells (**Figure S2**), they were used as exposure variables in multivariable MR analysis. The causal relationship between eosinophil count and psoriasis persisted (OR: 1.375, 95% CI: 1.045–1.808) even considering the association (**Figure 3D**). We further explored the correlation between eosinophil counts and the severity of psoriasis in the Chinese population. All three assessment methods showed a positive correlation, with the PASI score showing a strongest correlation (*P* = 6.6×10^-5^) (**Figure 3E**). This indicated that eosinophils have a potential impact on the severity of psoriasis.

### 3.4 The role of NLR, PLR, and LMR in psoriasis

NLR, PLR, and LMR, which are calculated using white blood cell count, have been proven to be important indices in inflammatory diseases (50–52). Whether they can be used to evaluate psoriasis remains unclear. Therefore, the relationship between these three indices and psoriasis was explored further.

**Figure 4:**
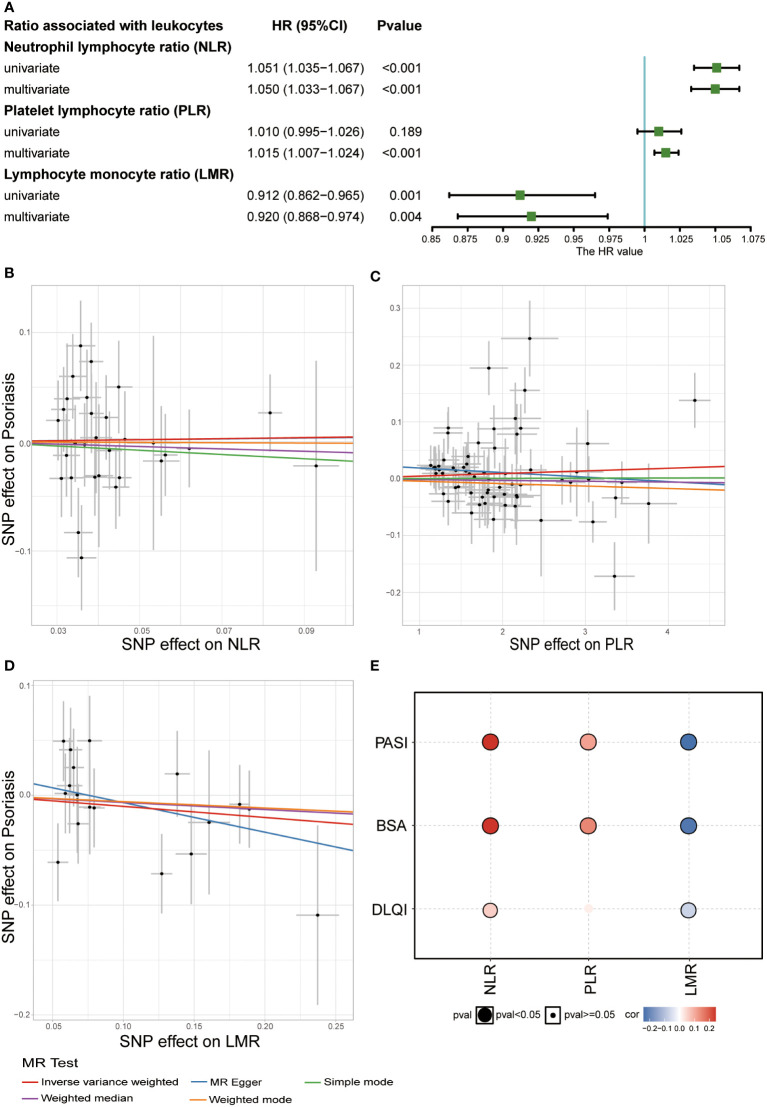
Relationship between white blood cell ratios (NLR, PLR, and LMR) and psoriasis. **(A)** Result of observational study about white blood cell ratios (NLR, PLR, and LMR) and psoriasis in UKB (Covariates included sex, age, BMI, smoking status, and alcohol drinking status and three ratios in multivariate Cox regression). **(B)** Scatter plot of MR analysis related to NLR and psoriasis. **(C)** Scatter plot of MR analysis related to PLR and psoriasis. **(D)** Scatter plot of MR analysis related to LMR and psoriasis. **(E)** Correlation between the three ratios and severity of psoriasis (Spearman’s correlation).

Their association with psoriasis was demonstrated in the UKB dataset by constructing a Cox regression model. In the univariate Cox regression model, NLR (HRs: 1.051, 95% CIs: 1.035–1.067) was a potential risk factor, whereas LMR showed a protective function in psoriasis (HRs: 0.912, 95% CIs: 0.862–0.965). To remove the impact of confounding factors, we adjusted for sex, age, BMI, smoking status, and alcohol drinking status in the multivariate Cox regression model, and found that high PLR might increase the risk of psoriasis (HRs: 1.015, 95% CIs: 1.007–1.024) (**Figure 4A**).

To explore the causal relationship between these three ratios and psoriasis, we performed a two-sample MR analysis. Due to the rarity of common large-scale GWAS on these three indices, GWAS analysis was performed in the UKB dataset. By setting the threshold of the *P* value, we discovered 5973 SNPs associated with NLR, 15473 SNPs associated with PLR, and 4606 SNPs associated with LMR (*P* adjusted by false discovery rate <8.31 × 10^-9^) (**Figure S4** and **Table S8**). Our GWAS results, combined with GWAS summary data related to psoriasis from the FinnGen biobank, were used to perform two-sample MR analysis. No causal relationship was observed between the three indices and psoriasis (**Figures 4B-D** and **Table S9**). Subsequently, we studied the correlation between these three indices and psoriasis to explore their impact on the severity of psoriasis in the Chinese population. NLR and PLR positively correlated with the severity of psoriasis, particularly PASI score, and BSA, whereas LMR negatively correlated with the severity of psoriasis (**Figure 4E**). This indicated that the three indices might be potential predictive indices for the severity of psoriasis.

## 4 Discussion

This study revealed an association between circulating white blood cells and psoriasis in two large-scale populations. One-sample MR combined with two-sample MR analysis was performed in our study, making our results more reliable. Eosinophils showed a definite causal relationship with psoriasis. In addition, the three indices, NLR, PLR, and LMR, were further verified to play important roles in psoriasis. Our study revealed an explicit causal relationship between eosinophils and psoriasis, and provided new insights into the research and clinical practice of psoriasis care.

The observational study and MR analysis revealed that circulating white blood cells are associated with psoriasis. In particular, we found a causal relationship between eosinophils and psoriasis, and eosinophils positively correlated with psoriasis severity. Currently, psoriasis is considered an inflammatory skin disease mediated by T helper 1/17 cells, and the IL-17/IL-23 axis has been regarded as a key driver of psoriasis pathogenesis (53). Eosinophils are involved in type II immune response, which is related to T helper 2 cells and various interleukins (including IL-4, IL-5, IL-9, IL-13, IL-31, and IL-33, among others), differing from the IL-17/23 axis of psoriasis (54). Cytokines related to type II immune response, such as IL-4, could suppress the IL-17/23 axis of psoriasis in lesional skin (55). Furthermore, Ghoreschi et al. reported that IL-4 therapy could improve psoriasis (56). Nevertheless, several studies also observed a significant increase in eosinophils and cytokines, such as IL-4, IL-5, IL-9, IL-31, and IL-33, among others, in the blood of patients with psoriasis (7, 57–60). This contradiction might be caused by the location and environment of these immune cells and cytokines. Herein, a potential association between psoriasis and allergic diseases related to type II immune response, such as asthma and atopic dermatitis, was further verified in the UKB participants (chi-squared test, both P <0.05) (**Table S10**). Thus, type II immune response may play an important role in psoriasis; however, this role remains unclear.

In addition to type II immune response, eosinophils might have an effect on psoriasis *via* other mechanisms. In clinical practice, eosinophils are associated with pruritus, and over 85% of psoriasis patients suffer from pruritus (61). Persistent pruritus may lead to more scratching in the lesional skin of psoriasis patients, which may further aggravate psoriasis (62). Moreover, Kim et al. reported that eosinophils provide inflammatory signals that accelerate psoriasis pathogenesis (63). TLR7 is expressed in eosinophils and regulates the secretion of inflammatory mediators, thereby promoting the migration, activation, and survival of neutrophils in psoriasis, which provides a possible mechanism that explains the causal relationship between eosinophils and psoriasis (63). In general, eosinophils play an important role in psoriasis; however, the specific mechanism needs to be further explored.

Besides eosinophils, the other four cell types showed unique profiles in psoriasis. Both neutrophils and monocytes presented strong risk factors for psoriasis, which is consistent with the results of previous studies (9, 64). Neutrophils in psoriatic skin lesions serve as a typical histopathological hallmark of psoriasis, and they could also release IL-17 and are involved in the inflammatory cascade in psoriatic skin lesions (65). The respiratory burst with reactive oxygen species generation, degranulation, and the formation of neutrophil extracellular traps from neutrophils have been discovered to contribute to the immunopathogenesis of psoriasis (10). Monocytes showed a significant increase in chemotactic response in psoriasis (66), and also produce some cytokines of major importance in psoriasis, such as IL-1, IL-6, and tumor necrosis factors, among others (67). Immune cells derived from monocytes, such as dendritic cells and macrophages, play a crucial role in psoriasis-like inflammation (68, 69) These immune cells can secrete drivers of inflammation, such as IL-23, to promote the incidence of psoriasis (70). Meanwhile, biological therapy for psoriasis could decrease the activity of monocytes and neutrophils (71). Although our cohort study also highlighted the importance of these two cell types, no sufficiently causal relationship between them and psoriasis was identified in the MR results. This discrepancy might be explained by epidemiological confounding factors related to the study cohort. In addition, other studies have reported that immune cells derived from the skin might also play a crucial role in the incidence of psoriasis (65, 67); hence, the causal relationship between these two cell types in skin lesions and psoriasis may need to be explored in the future. Although lymphocytes have been proven to be a key factor in the pathogenesis of psoriasis (3, 72), the circulating lymphocyte count tends to decrease in patients with psoriasis, according to a previous study (15). One possible hypothesis is that numerous lymphocytes flow into the skin from the peripheral blood, which causes a decrease in circulating lymphocytes in patients with psoriasis (73). In our study, lymphocytes did not show a significant causal relationship with psoriasis; however, it should be noted that different subtypes of circulating lymphocytes may undergo diverse changes, which requires further study. Basophils, as a type of immune cells associated with allergies, have been reported to be involved in the regulation of skin inflammation (74) and to play an important role in psoriasis-associated pruritus (75). Due to the rarity of basophils in the blood and skin, their function in psoriasis was often ignored in previous studies and is still unclear. Our results showed that basophils may have a potential causal association with psoriasis. However, the statistical significance of the finding could not be substantiated because the count values of basophils in most participants were zero (**Figure S1**). More data are required to verify the relationship between basophils and psoriasis.

NLR, PLR, and LMR are markers of systemic inflammatory response and showed significant changes in patients with psoriasis (7, 15, 64, 76). However, these studies were limited by their small sample sizes. Moreover, large-scale GWAS data associated with these three indices are unavailable. Our study used large-scale population data to verify this conclusion and provide a potential genetic variation related to these three indices. The MR analysis showed no evidence of a causal relationship between psoriasis and these indices. These changes in psoriasis may be caused by inflammation itself, rather than a direct causal relationship. However, it is certain that these three indices are still potential predictors of the incidence and severity of psoriasis.

Although our study showed an important relationship between circulating white blood cells and psoriasis in a large-scale population, there were some limitations. First, our cohort study and MR analysis were only based on a European population. Thus, the proven causal relationship may only apply to the European population, and further investigation is needed in other races. Second, white blood cells were divided into five main types in our study; however, these subtypes of white blood cells could be further subdivided. For example, lymphocytes can be further divided into B cells and T cells. Identification of key cell subpopulations in psoriasis may be challenging because of their complex cell composition. Third, we identified an association between circulating white blood cells and psoriasis; however, the specific mechanism of circulating white blood cells needs to be further explored. Finally, the LD score regression intercepts of PLR in the GWAS were relatively high, indicating that a more refined population division is needed for the Caucasian population of the UKB.

In summary, our study identified a definite association between circulating white blood cells and psoriasis, which can be helpful in dermato-epidemiology and clinical practice to an extent. The incidence of psoriasis is an important public health concern, and as test results from peripheral blood, white blood cell count is relatively easy to obtain for the prediction of psoriasis risk. Eosinophils, NLR, PLR, and LMR might be indices used to evaluate treatment effects due to their correlation with the severity of psoriasis. Eosinophils might also become a novel target in psoriasis treatment; however, more basic research needs to be performed to elaborate on the specific mechanism of eosinophils in psoriasis.

## Data availability statement

The original contributions presented in the study are included in the article/**Supplementary Material**. Further inquiries can be directed to the corresponding authors.

## Ethics statement

The studies involving human participants were reviewed and approved by The North West Multi-Center Research Ethics Committee and the institutional research ethics boards of Xiangya Hospital. The patients/participants provided their written informed consent to participate in this study.

## Author contributions

GWZ and XMR designed the experiments, analyzed the data, and prepared the manuscript. HL, MXS, GXZ, XC, and FYM provided supervision and oversaw final manuscript preparation. ZWT, WL, and WQC collected the data and helped with observational study. YH, BLW, and HLZ cleaned the data and helped with MR analysis. All the authors contributed to and approved the final version of this manuscript.
