# Supplementary material for: Exploring the association and causal effect between white blood cells and psoriasis using large-scale population data
Source: Front Immunol. 2023 Feb 14;14:1043380. doi: 10.3389/fimmu.2023.1043380 (PMC9971993; doi:10.3389/fimmu.2023.1043380)
Supplement: Supplementary file 2 [file Image_1.pdf]

Fig .S1

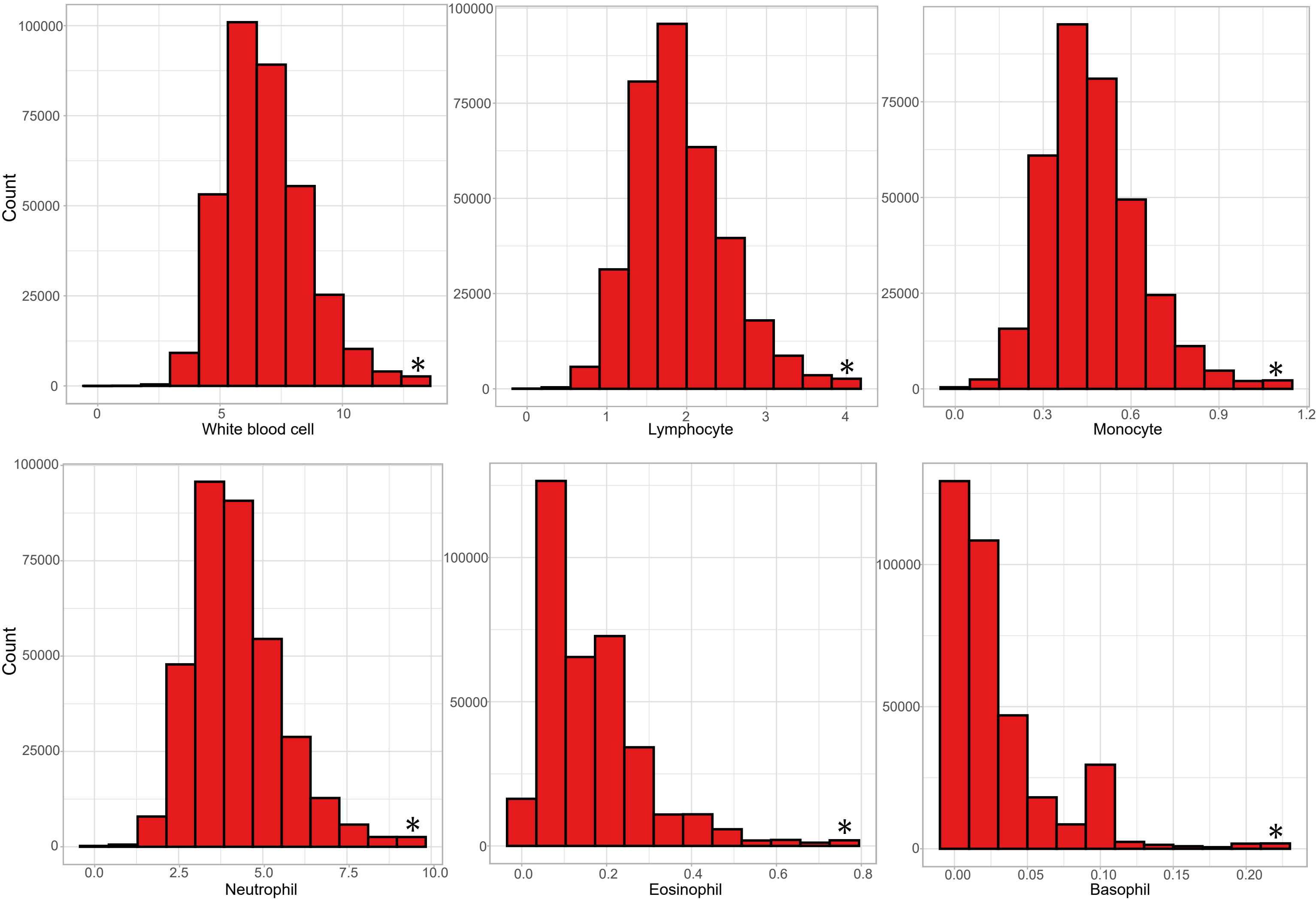
Count
100000
75000
50000
25000
0
0
5
10
White blood cell
100000
75000
50000
25000
0
0
1
2
3
4
Lymphocyte
75000
50000
25000
0
0.0
0.3
0.6
0.9
1.2
Monocyte
Count
100000
75000
50000
25000
0
0.0
2.5
5.0
7.5
10.0
Neutrophil
100000
50000
0
0.0
0.2
0.4
0.6
0.8
Eosinophil
100000
50000
0
0.00
0.05
0.10
0.15
0.20
Basophil
*

Fig. S2

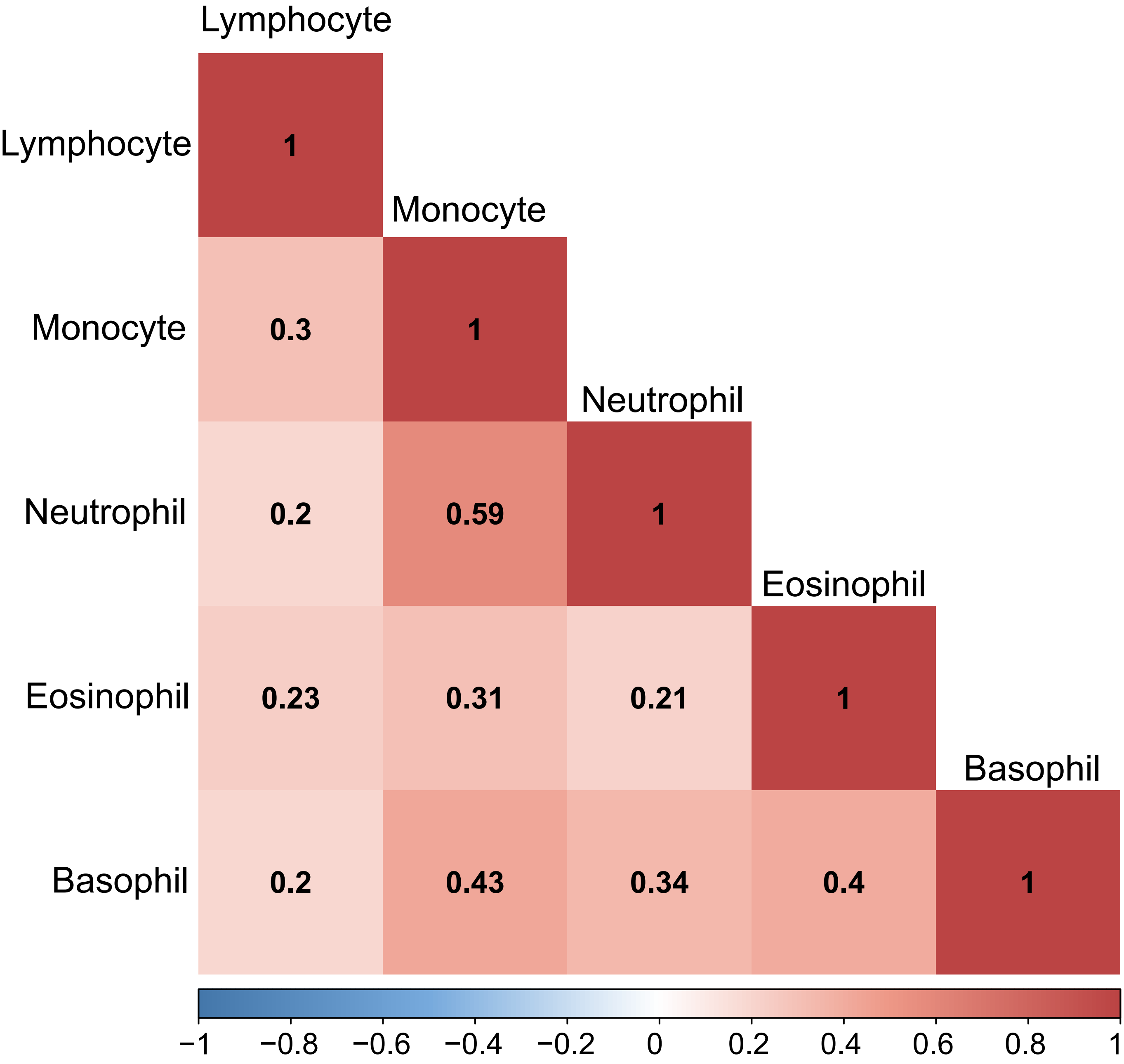
Lymphocyte
Monocyte
Neutrophil
Eosinophil
Basophil
1
0.3
1
0.2
0.59
1
0.23
0.31
0.21
1
0.2
0.43
0.34
0.4
1
−1
−0.8
−0.6
−0.4
−0.2
0
0.2
0.4
0.6
0.8
1

Fig. S3

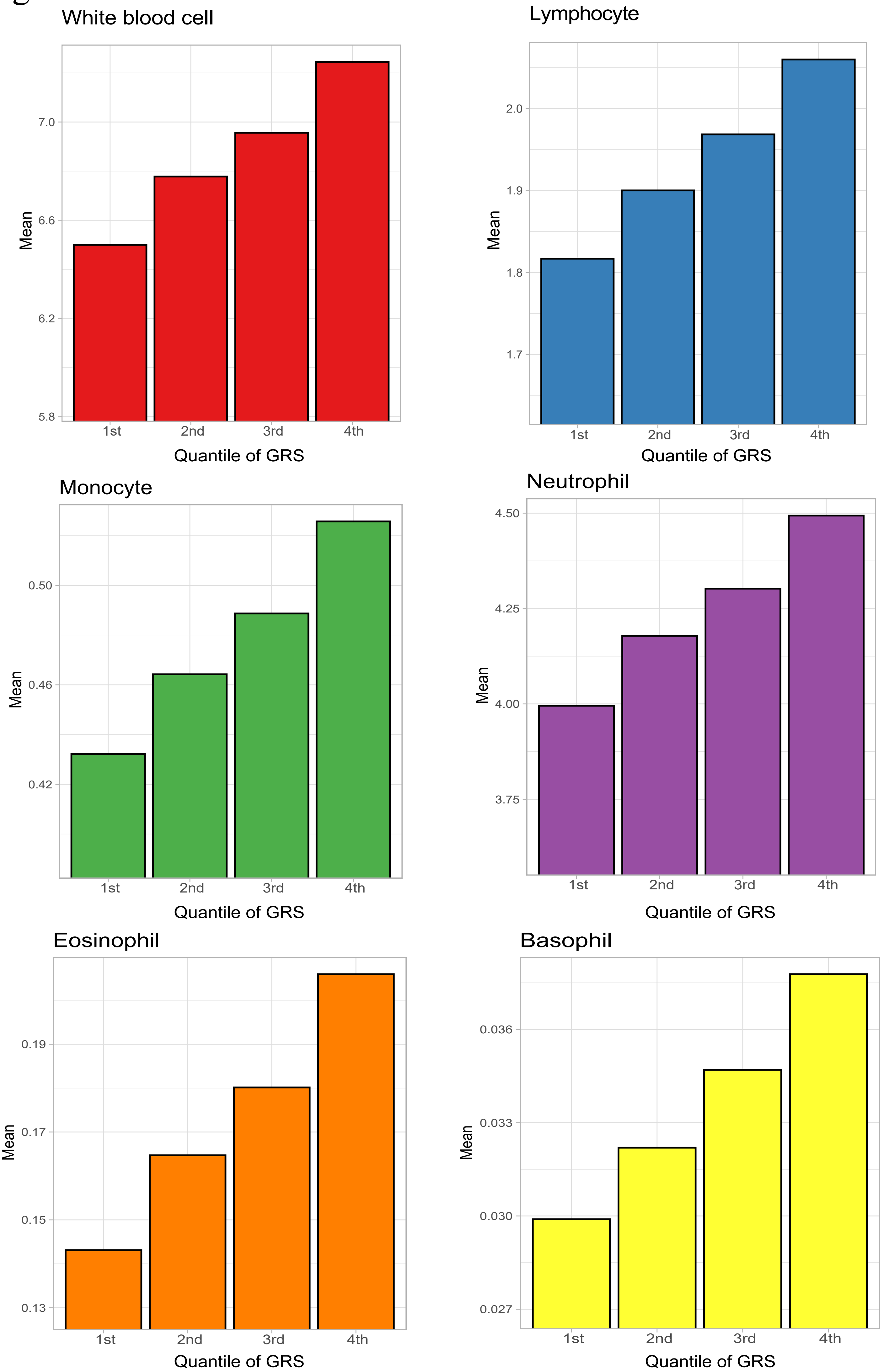

White blood cell
Mean
7.0
6.6
6.2
5.8
1st
2nd
3rd
4th
Quantile of GRS
Lymphocyte
Mean
2.0
1.9
1.8
1.7
1st
2nd
3rd
4th
Quantile of GRS
Monocyte
Mean
0.50
0.46
0.42
1st
2nd
3rd
4th
Quantile of GRS
Neutrophil
Mean
4.50
4.25
4.00
3.75
1st
2nd
3rd
4th
Quantile of GRS
Eosinophil
Mean
0.19
0.17
0.15
0.13
1st
2nd
3rd
4th
Quantile of GRS
Basophil
Mean
0.036
0.033
0.030
0.027
1st
2nd
3rd
4th
Quantile of GRS

Fig. S4

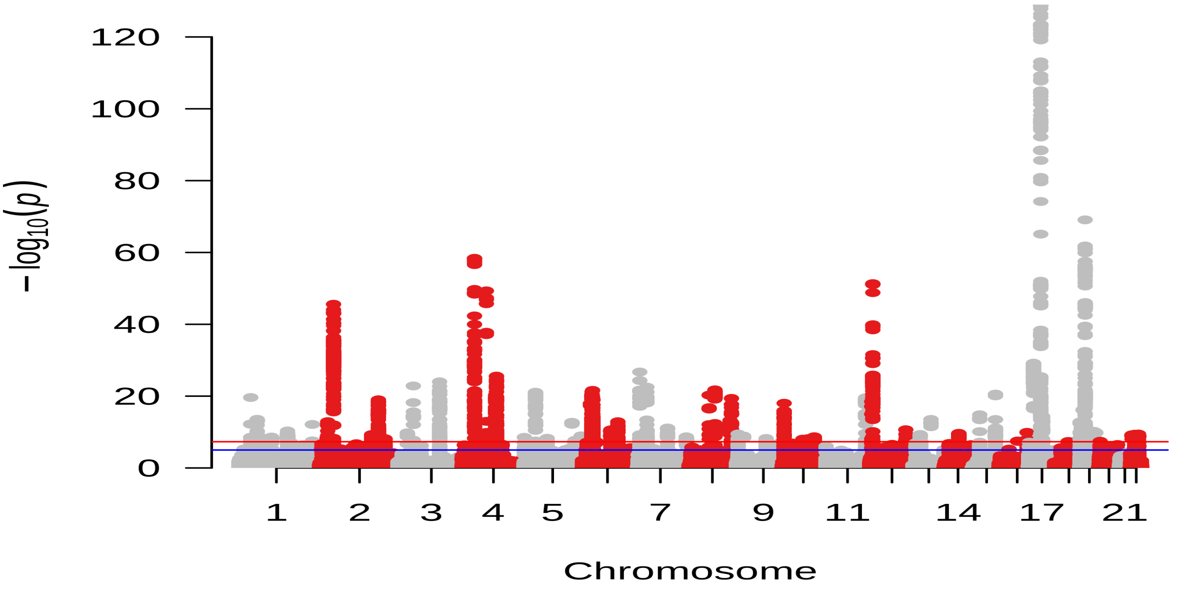

120
100
80
60
40
20
0
$-\log_{10}(p)$
1
2
3
4
5
7
9
11
14
17
21
Chromosome

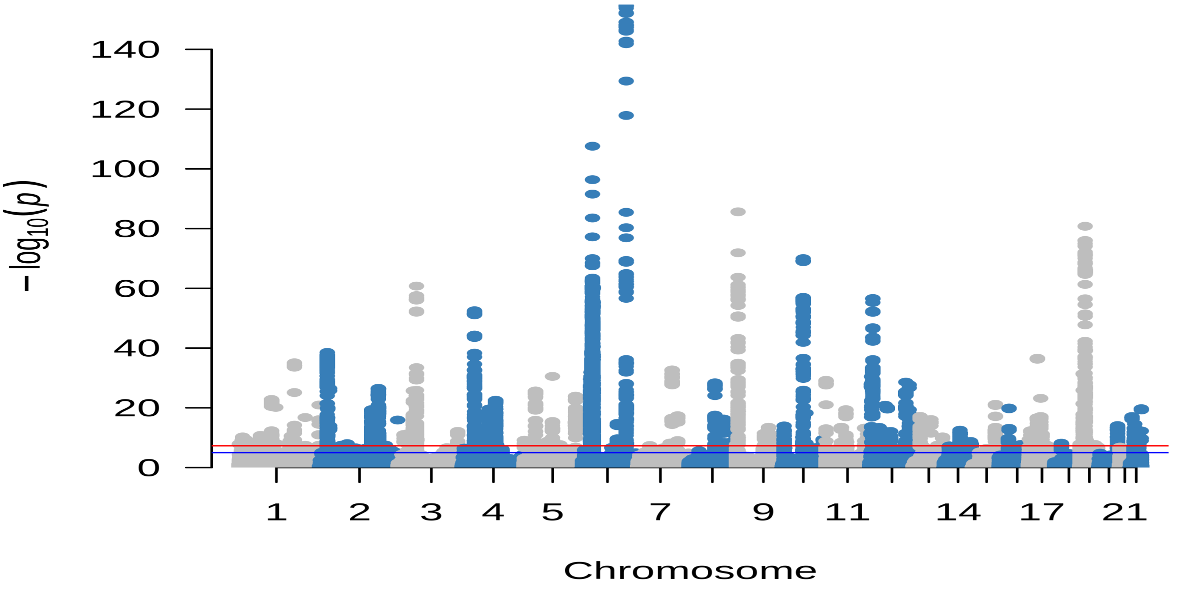

140
120
100
80
60
40
20
0
$-\log_{10}(p)$
1
2
3
4
5
7
9
11
14
17
21
Chromosome

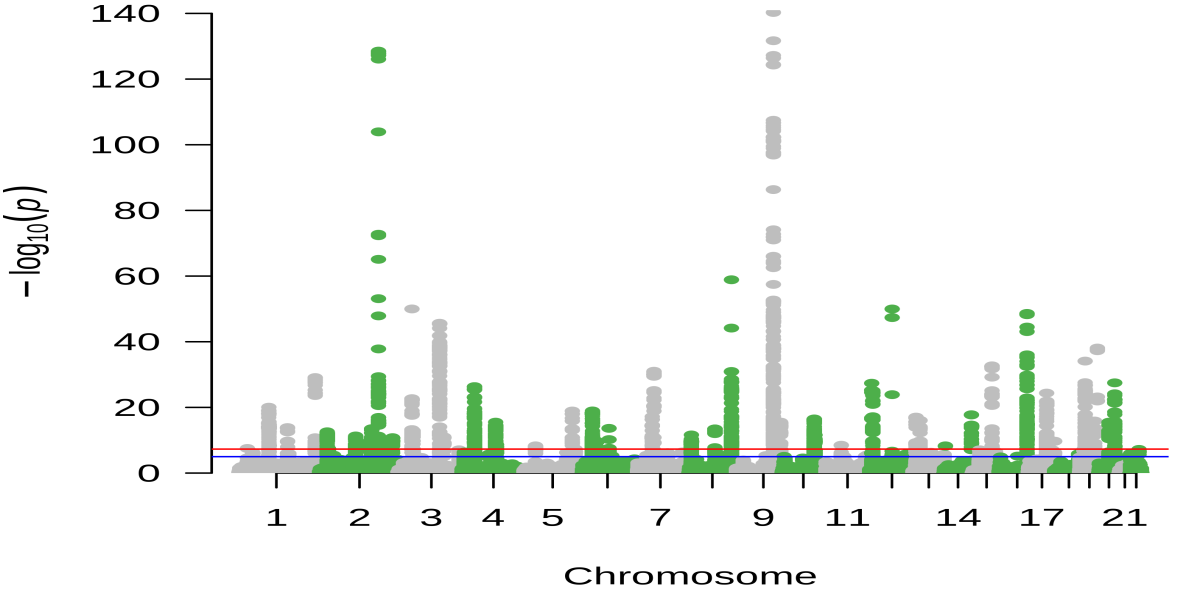

140
120
100
80
60
40
20
0
$-\log_{10}(p)$
1
2
3
4
5
7
9
11
14
17
21
Chromosome

Fig. S5

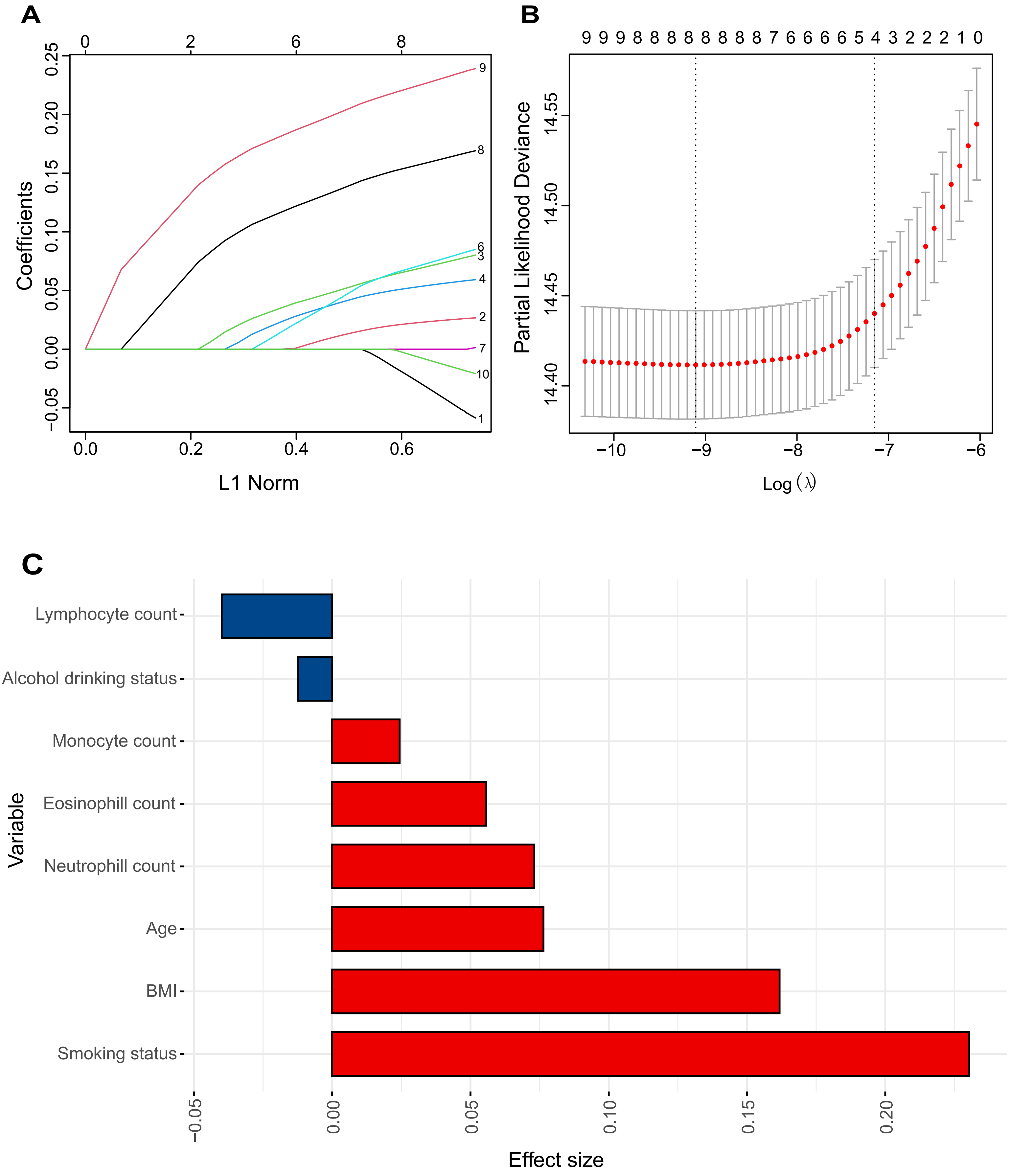
A
Coefficients
L1 Norm
B
Partial Likelihood Deviance
Log (λ)
C
Lymphocyte count
Alcohol drinking status
Monocyte count
Eosinophill count
Neutrophill count
Age
BMI
Smoking status
Variable
Effect size

Fig. S6

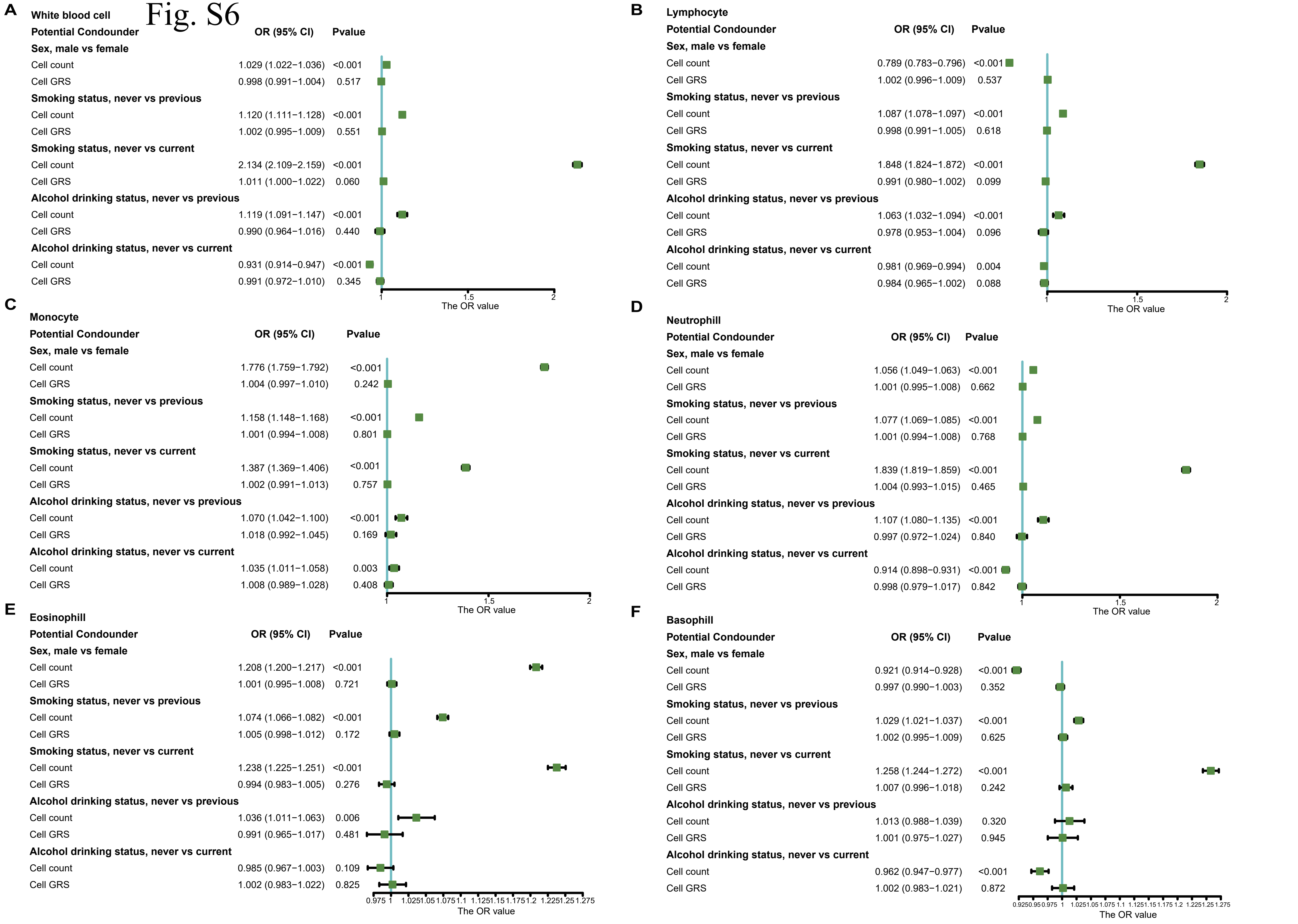
A
White blood cell
Potential Condounder
OR (95% CI)
Pvalue
Sex, male vs female
Cell count 1.029 (1.022−1.036) <0.001
Cell GRS 0.998 (0.991−1.004) 0.517
Smoking status, never vs previous
Cell count 1.120 (1.111−1.128) <0.001
Cell GRS 1.002 (0.995−1.009) 0.551
Smoking status, never vs current
Cell count 2.134 (2.109−2.159) <0.001
Cell GRS 1.011 (1.000−1.022) 0.060
Alcohol drinking status, never vs previous
Cell count 1.119 (1.091−1.147) <0.001
Cell GRS 0.990 (0.964−1.016) 0.440
Alcohol drinking status, never vs current
Cell count 0.931 (0.914−0.947) <0.001
Cell GRS 0.991 (0.972−1.010) 0.345
1
1.5
2
The OR value
B
Lymphocyte
Potential Condounder
OR (95% CI)
Pvalue
Sex, male vs female
Cell count 0.789 (0.783−0.796) <0.001
Cell GRS 1.002 (0.996−1.009) 0.537
Smoking status, never vs previous
Cell count 1.087 (1.078−1.097) <0.001
Cell GRS 0.998 (0.991−1.005) 0.618
Smoking status, never vs current
Cell count 1.848 (1.824−1.872) <0.001
Cell GRS 0.991 (0.980−1.002) 0.099
Alcohol drinking status, never vs previous
Cell count 1.063 (1.032−1.094) <0.001
Cell GRS 0.978 (0.953−1.004) 0.096
Alcohol drinking status, never vs current
Cell count 0.981 (0.969−0.994) 0.004
Cell GRS 0.984 (0.965−1.002) 0.088
1
1.5
2
The OR value
C
Monocyte
Potential Condounder
OR (95% CI)
Pvalue
Sex, male vs female
Cell count 1.776 (1.759−1.792) <0.001
Cell GRS 1.004 (0.997−1.010) 0.242
Smoking status, never vs previous
Cell count 1.158 (1.148−1.168) <0.001
Cell GRS 1.001 (0.994−1.008) 0.801
Smoking status, never vs current
Cell count 1.387 (1.369−1.406) <0.001
Cell GRS 1.002 (0.991−1.013) 0.757
Alcohol drinking status, never vs previous
Cell count 1.070 (1.042−1.100) <0.001
Cell GRS 1.018 (0.992−1.045) 0.169
Alcohol drinking status, never vs current
Cell count 1.035 (1.011−1.058) 0.003
Cell GRS 1.008 (0.989−1.028) 0.408
1
1.5
2
The OR value
D
Neutrophill
Potential Condounder
OR (95% CI)
Pvalue
Sex, male vs female
Cell count 1.056 (1.049−1.063) <0.001
Cell GRS 1.001 (0.995−1.008) 0.662
Smoking status, never vs previous
Cell count 1.077 (1.069−1.085) <0.001
Cell GRS 1.001 (0.994−1.008) 0.768
Smoking status, never vs current
Cell count 1.839 (1.819−1.859) <0.001
Cell GRS 1.004 (0.993−1.015) 0.465
Alcohol drinking status, never vs previous
Cell count 1.107 (1.080−1.135) <0.001
Cell GRS 0.997 (0.972−1.024) 0.840
Alcohol drinking status, never vs current
Cell count 0.914 (0.898−0.931) <0.001
Cell GRS 0.998 (0.979−1.017) 0.842
1
1.5
2
The OR value
E
Eosinophill
Potential Condounder
OR (95% CI)
Pvalue
Sex, male vs female
Cell count 1.208 (1.200−1.217) <0.001
Cell GRS 1.001 (0.995−1.008) 0.721
Smoking status, never vs previous
Cell count 1.074 (1.066−1.082) <0.001
Cell GRS 1.005 (0.998−1.012) 0.172
Smoking status, never vs current
Cell count 1.238 (1.225−1.251) <0.001
Cell GRS 0.994 (0.983−1.005) 0.276
Alcohol drinking status, never vs previous
Cell count 1.036 (1.011−1.063) 0.006
Cell GRS 0.991 (0.965−1.017) 0.481
Alcohol drinking status, never vs current
Cell count 0.985 (0.967−1.003) 0.109
Cell GRS 1.002 (0.983−1.022) 0.825
0.975 1 1.025 1.05 1.075 1.1 1.125 1.15 1.175 1.2 1.225 1.25 1.275
The OR value
F
Basophill
Potential Condounder
OR (95% CI)
Pvalue
Sex, male vs female
Cell count 0.921 (0.914−0.928) <0.001
Cell GRS 0.997 (0.990−1.003) 0.352
Smoking status, never vs previous
Cell count 1.029 (1.021−1.037) <0.001
Cell GRS 1.002 (0.995−1.009) 0.625
Smoking status, never vs current
Cell count 1.258 (1.244−1.272) <0.001
Cell GRS 1.007 (0.996−1.018) 0.242
Alcohol drinking status, never vs previous
Cell count 1.013 (0.988−1.039) 0.320
Cell GRS 1.001 (0.975−1.027) 0.945
Alcohol drinking status, never vs current
Cell count 0.962 (0.947−0.977) <0.001
Cell GRS 1.002 (0.983−1.021) 0.872
0.925 0.95 0.975 1 1.025 1.05 1.075 1.1 1.125 1.15 1.175 1.2 1.225 1.25 1.275
The OR value

Fig. S7

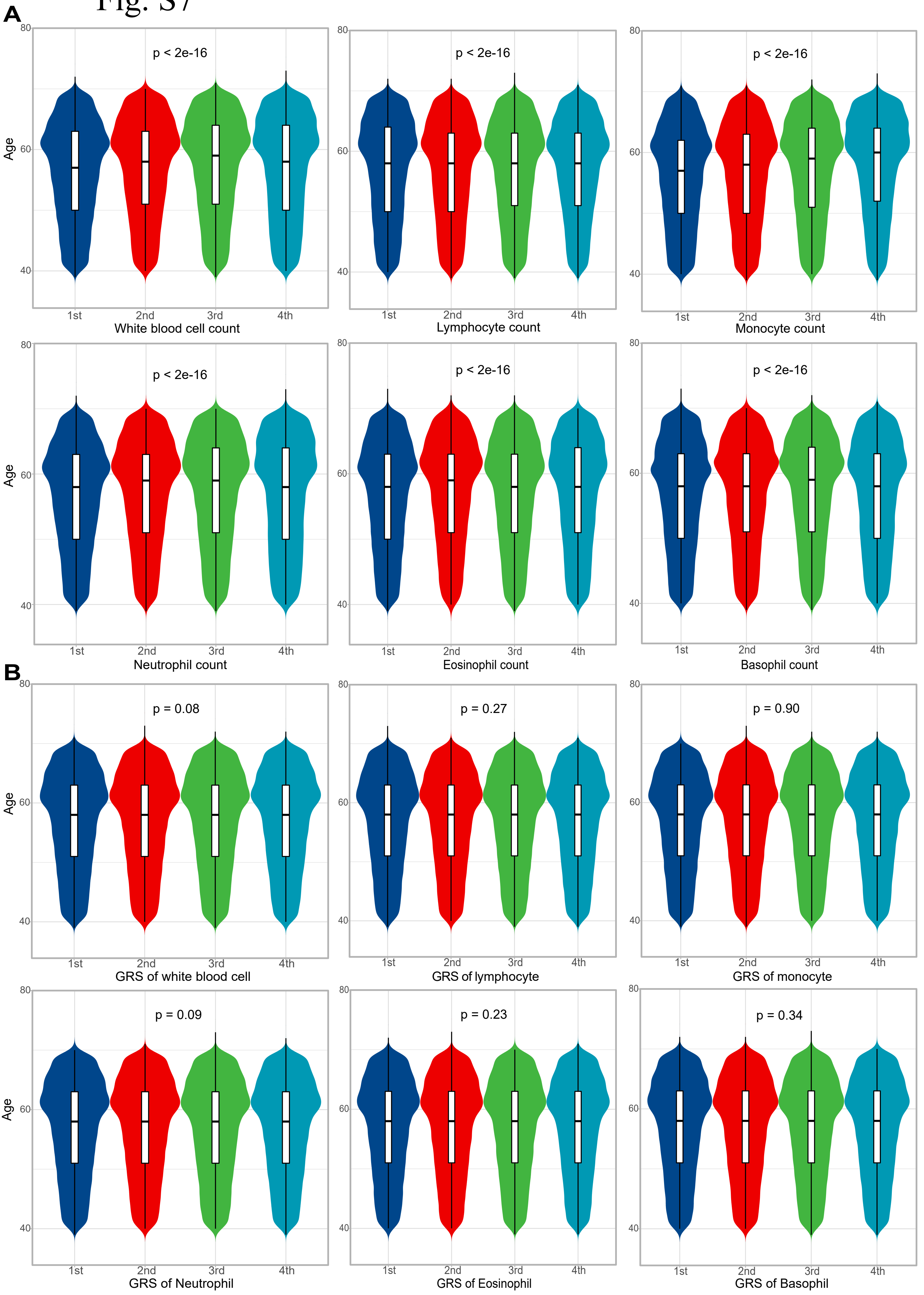

A
B
Age
p < 2e-16
p = 0.08
p = 0.27
p = 0.90
p = 0.09
p = 0.23
p = 0.34
1st
2nd
3rd
4th
White blood cell count
Lymphocyte count
Monocyte count
Neutrophil count
Eosinophil count
Basophil count
GRS of white blood cell
GRS of lymphocyte
GRS of monocyte
GRS of Neutrophil
GRS of Eosinophil
GRS of Basophil

Fig. S8

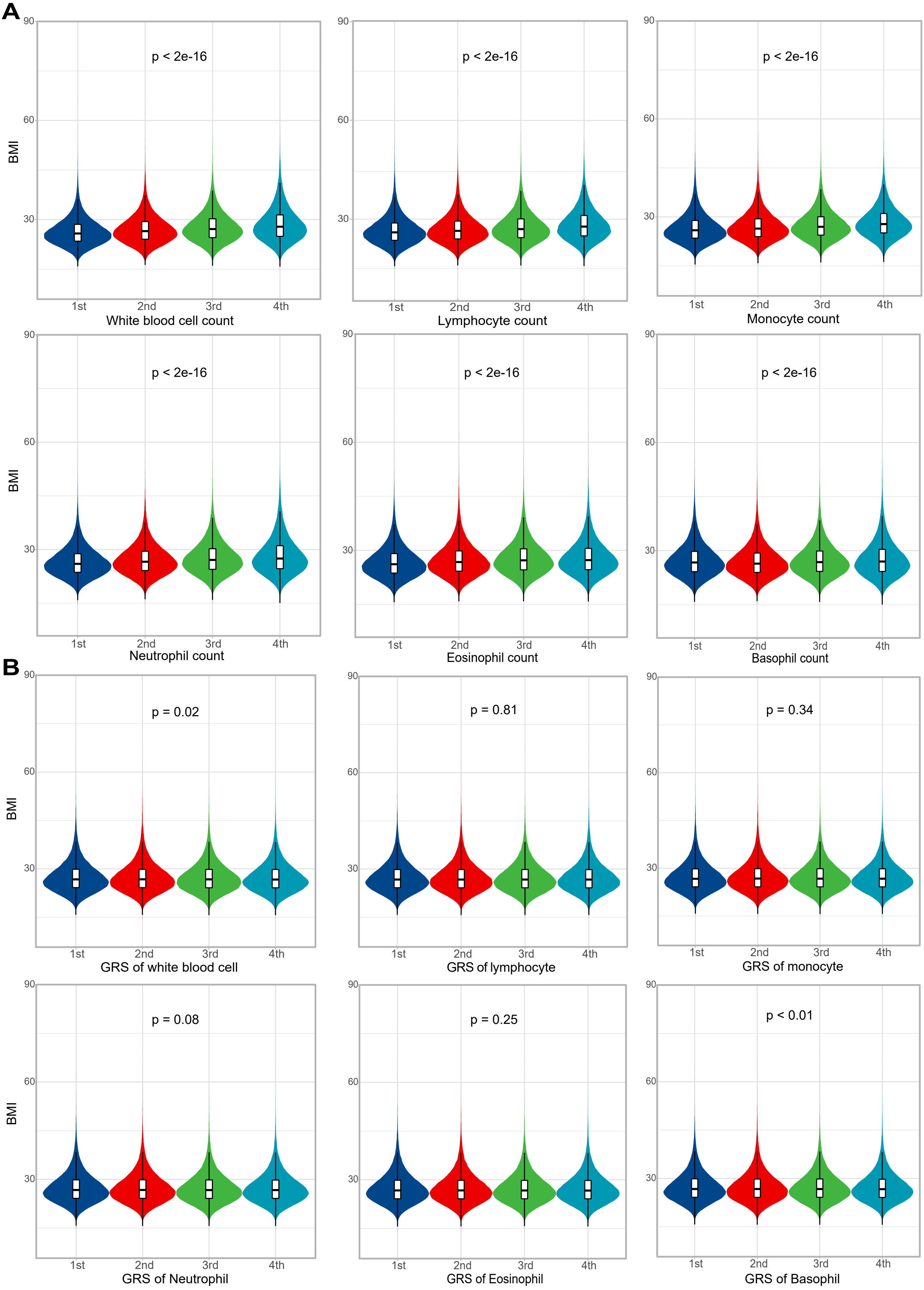
A
B
BMI
90
60
30
p < 2e-16
1st
2nd
3rd
4th
White blood cell count
Lymphocyte count
Monocyte count
Neutrophil count
Eosinophil count
Basophil count
p = 0.02
p = 0.81
p = 0.34
p = 0.08
p = 0.25
p < 0.01
GRS of white blood cell
GRS of lymphocyte
GRS of monocyte
GRS of Neutrophil
GRS of Eosinophil
GRS of Basophil

Supplementary Figure Legends

Figure S1. The distribution of white blood cell count in UKB. (* represented that bar included 0.5% subjects of the highest level of white blood cell count).

Figure S2. Correlation among different types of white blood cells in UKB. Values in heat map represented the correlation coefficients. Correlation was calculated by Spearman's correlation, and all $P < 2.2\times10^{-16}$.

Figure S3. The mean count of different white blood cells in each category of GRSs associated with leukocytes.

Figure S4. Manhattan plot of three ratios in GWAS study. Red: NLR; Blue: PLR; Green: LMR; blue and red lines respectively represent threshold values of 10^{-5} and 10^{-8}.

Figure S5. Lasso regression of white blood cells and psoriasis in UKB. (A) The change in coefficients of each independent variable with change of L1 norm; **(B)** Relationship between logarithmic value of Lambda and error (The dotted lines were the range of Lambda that can be selected); **(C)** Effect size of each independent variable after lasso regression.

Figure S6. The association between confounding factors and the count or GRSs of white blood cells in logistic regression models. (A)-(F) represented different features of white blood cells, and confounding factors included sex, smoking status and alcohol drinking status.

Figure S7. The association between age and the count or GRSs of white blood cells. (A) the count of white blood cells; **(B)** the GRSs of white blood cells; statistical test: Kruskal-Wallis test.

Figure S8. The association between BMI and the count or GRSs of white blood cells. (A) the count of white blood cells; **(B)** the GRSs of white blood cells; statistical test: Kruskal-Wallis test.

Supplementary Table Legends

Table S1. The main Data-Fields of variables in UKB.

Table S2. The meta-GWAS information to calculate GRSs in UKB. A1 represented effector allele, A2 represented reference allele.

Table S3. The information of overlapping SNPs between basophil count and BMI from PhenoScanner.

Table S4. Sensitivity analysis of cohort study in UKB.

Table S5. Association between white blood cells and psoriasis in logistic regression.

Table S6. Two-sample MR analyses testing effects of the count of white blood cells subtypes on psoriasis.

Table S7. Leave-one-out analysis presenting IVW causal estimates testing effect of white blood cell and eosinophil count on psoriasis.

Table S8. SNPs associated with NLR, PLR and LMR through GWAS analysis in UKB. A1 represented effector allele, A2 represented reference allele.

Table S9. Two-sample MR analyses testing effects of ratios associated with white blood cells on psoriasis.

Table S10. The association between allergic diseases and psoriasis in UKB. Patients with asthma and atopic dermatitis were from primary care, hospital admission, self-report and others in baseline from UKB. The method of statistical analysis was Chi-squared test.
